# Translational Neuroimaging of the Mood and Anxiety Disorders

**DOI:** 10.1155/2014/240769

**Published:** 2014-05-12

**Authors:** Su Lui, Qiyong Gong, Yong He, Georg Northoff, John A. Sweeney

**Affiliations:** ^1^Huaxi MR Research Center (HMRRC), Department of Radiology, Psychiatry and Neurology, West China Hospital of Sichuan University, Chengdu, Sichuan 610041, China; ^2^State Key Laboratory of Cognitive Neuroscience and Learning, Beijing Normal University, Beijing 100875, China; ^3^Unit of Mind, Brain Imaging and Neuroethics, University of Ottawa Institute of Mental Health Research, Ottawa, ON, Canada K1Z 7K4; ^4^Departments of Psychiatry and Pediatrics, University of Texas Southwestern Medical Center, Dallas, TX 75390-9086, USA

Mood and anxiety disorders include all types of depression, bipolar disorders, and anxiety disorders and are among the top 10 causes of disability worldwide. There is no effective diagnosis or treatment mainly due to our poor understanding of the underlying pathophysiology and particularly the lack of objective biomarkers for diagnosis and prognosis. In recent years, researchers have made remarkable advances in understanding the cerebral deficits in patients with mood andanxiety disorders using various brain imaging methods such as computed tomography (CT), magnetic resonance imaging (MRI), and positron emission tomography (PET). Particularly over the past two decades, with the development of novel neuroimaging methods (e.g., structural MRI, functional MRI, diffusion MRI, and EEG/MEG), researchers have made great strides forward in the understanding of both anatomical and functional cerebral deficits in patients with mood andanxiety disorders.

To accelerate the process of translating these new techniques to clinical applications, a number of important issues must be addressed such as their ability to consistently detect characteristic cerebral deficits in individuals with mood andanxiety disorders, the relationships of cerebral deficits to clinical symptoms and genetic characteristics, and the degree to which the deficits respond to different therapies. In this special issue, several research groups report findings that address some of these issues.

The paper by X. Wang et al. addresses the role of self-perspective in reappraisal process of depressed patients. With functional MRI, the authors found that impaired modulatory effects of amygdala in depressed patients are compensated for by increased utilization of cognitive control resources, with dissociable effects for different self-perspectives in reappraisal. The study by S. Deppermann et al. used functional near-infrared spectroscopy (fNIRS) to monitor the treatment effect of repetitive transcranial magnetic stimulation (rTMS) on panic disorder (PD). Their findings support that PD is characterized by prefrontal hypoactivation during cognitive performance. The study by T.-Y. Liu et al. investigated the cortical abnormalities of early emotion perception in patients with major depressive disorder (MDD) and bipolar disorder (BD) using magnetoencephalography (MEG), which showed the potential of MEG measurements in separating MDD from BD.

The study by K. Hilbert et al. used fMRI to investigate the influence of stimulus modality on neural fear processing in dental phobia, which enlarges our knowledge about neural correlates of phobias. The paper by N. Jaworska et al. assessed cortical thickness using high resolution MRI in patients with varying ages of MDD onset and trauma history. Their findings demonstrate that anatomical brain deficits can be expressed differently in relation to age of onset of depression and the presence of childhood trauma. The paper by B. E. Depue et al. used MRI to determine whether comorbid posttraumatic stress disorder (PTSD) and mild traumatic brain injury (mTBI) are characterized by altered brain structure in the same regions as has been observed when PTSD or TBI is present without the other condition using both voxel-based morphometry and surface-based morphometry. These are highly comorbid conditions, and thus this study addresses an important clinical question. Their findings suggest that alterations in brain anatomy in veterans with comorbid PTSD/mTBI are associated with both cognitive deficits and trauma symptoms related to PTSD. The study by C. Qiu et al. used diffusion tensor imaging (DTI) to characterize white-matter microstructural changes in patients with social anxiety disorder (SAD) and to describe their relationship to the severity of psychological symptoms. The study by H.-J. Li et al. employed a new index-surface-based regional homogeneity (ReHo) to analyze resting fMRI data in MDD patients, which may provide a new useful index to explore the pathophysiological mechanism of MDD. The paper by M. H. Serpa et al. evaluated the diagnostic performance of a neuroanatomical pattern classification method for discriminating psychotic MDD, bipolar I disorder (BD-I), and healthy controls using patients early in the course of illness. Though the findings are negative, perhaps related to the smaller sample size, this paper illustrates a very promising method for combining structural MRI data with other indices to characterize and differentiate individuals with mood disorders. The study by A. Sekiguchi et al. examined the white-matter integrity in 25 subjects 1 year after a Japanese earthquake to clarify the long-term effects of the severe psychological trauma on brain white matter. The study showed the potential ability of DTI in predicting the prognosis of physically healthy survivors after severe psychological trauma. Finally, the study by C. N. Kuswanto et al. examined the relationship between the* GRIN2B gene* and cerebral white-matter changes in BD, which is an example of an important line of work investigating relations between neuroimaging findings and genetic factors. Their findings enhance our understanding of the link between dysregulated glutamatergic neurotransmission and neuroimaging endophenotypes in BD.

In summary, the papers in this series highlight several important research strategies that are making it increasingly evident that the neuroimaging findings are of translational value for psychiatry. The results from these psychiatric neuroimaging studies not only help us to understand the pathogenesis of the mood and anxiety disorders but also show great potential to provide urgently needed objective biomarkers for clinical diagnosis and evaluation. Ultimately, psychiatric imaging may play an important role not only in differential diagnosis but also in monitoring the effects of therapeutic intervention on brain to advance drug discovery and clinical practice in the long run.

**Su Lui** **Qiyong Gong** **Yong He** **Georg Northoff** **John A. Sweeney**

